# Access and utilization of e-learning on tropical medicine at higher education institutions in Indonesia: A mixed-methods study

**DOI:** 10.1371/journal.pone.0335664

**Published:** 2025-12-31

**Authors:** Eti Poncorini Pamungkasari, Atik Maftuhah, Susan Dierickx, Bulan Kakanita Hermasari, Vitri Widyaningsih, Yusuf Ari Mashuri, Adaninggar Angesti Laras, Anis Sofia Harjanti, Nada Syifa Al Biruni, Utiya Nabila Maulani, Sami Alcedo, Elsa Herdiana Murhandarwati, Ari Probandari, Maria Zolfo

**Affiliations:** 1 Department of Public Health, Faculty of Medicine, Universitas Sebelas Maret, Surakarta, Indonesia; 2 Department of Medical Education, Faculty of Medicine, Universitas Sebelas Maret, Surakarta, Indonesia; 3 Department of Clinical Sciences, Institute of Tropical Medicine, Antwerp, Belgium; 4 Department of Parasitology, Faculty of Medicine, Universitas Sebelas Maret, Surakarta, Indonesia; 5 Research Group Medical and Health Profession Education, Faculty of Medicine, Universitas Sebelas Maret, Surakarta, Indonesia; 6 Center for Tropical Medicine, Faculty of Medicine, Public Health, and Nursing, Universitas Gadjah Mada, Indonesia; Kalasalingam Academy of Research and Education, INDIA

## Abstract

**Introduction:**

This study explored the experiences of medical students and lecturers with e-learning online resources on tropical medicine in Indonesia to find opportunities to enhance medical education.

**Methods:**

This was a mixed-methods study conducted between July and September 2022. The study involved academic staff and students from 13 universities in Indonesia. The universities were selected using stratified random sampling with consideration of accreditation level and geographical location. A survey with a structured self-reported questionnaire was conducted among 888 undergraduate and clinical medical students. For the qualitative study, 19 students and 12 academic staff participated in focus group discussions until data saturation was reached. Quantitative data were analysed descriptively, while thematic analysis was used to analyse the qualitative data.

**Results/discussion:**

Our study found that most of pre-clinical students never accessed a specific e-learning platform for tropical medicine (68.2%, n = 345), meanwhile, the majority of clinical students had ever accessed e-learning platforms (51.6%, n = 197) for tropical medicine, thus showing a statistically significant difference (p-value of <0.001) between both groups. The most used resources to learn tropical medicine consisted of online journals by both pre-clinical (58.7%, n = 297) and clinical students (74.1%, n = 283). Online journals were said to help students a lot to understand tropical medicine concepts because of the quality information they provide. Google Scholar was the most common search engine used to seek information. While there was not yet a specific e-learning platform for tropical medicine, both pre-clinical (88.8%, n = 409) and clinical students (73.0%, n = 279) hoped for the improvement of e-learning platform quality (p-value of 0.006) that had existed. Students have the perception that e-learning has a role as a learning tool that can support them, especially clinical students, to reach their competence in tropical medicine, even though there are barriers related to infrastructural aspects, including internet access and limited user-friendly and local platforms on tropical medicine.

**Conclusion:**

Preclinical and clinical students have the advantages of using e-learning on tropical medicine, even though there are some barriers related to access. However, specific e-learning platforms for tropical medicine are still very limited. Due to the importance of digital skills for the students as a medical professional in the future, there is an interest in developing a user-friendly e-learning platform specific for tropical medicine to facilitate medical students’ learning.

## Introduction

One of the strategies for easing the burden of health worker shortage and enhancing the delivery of high-quality medical education in low- and middle-income (LMICs) countries is through an effective e-learning system [1]. This is particularly important considering the significant challenges most LMICs face, including limited healthcare budgets, limited access to equipment and infrastructure, and an unfulfilled need for continuing professional development, including medical education programs. One way to provide effective and affordable education strategies is by investing in e-learning. The Lancet Global Commissioners for the Education of Professionals for the 21st Century stated, ‘The effect of electronic learning (e-learning) is likely to be revolutionary, although how precisely it will revamp professional education remains unknown [2].

The Government of Indonesia had started digitalizing learning materials in Higher Education before the COVID-19 pandemic [3], however in the unprecedented times of the COVID-19 pandemic, the medical education system is challenged to provide high-quality education, both in preclinical phases and in clinical rotations. Schools, colleges, and universities were temporarily forced to close [4,5]. As has happened in other countries, medical students in Indonesia cannot access their university libraries and laboratories, limiting their access to education and up-to-date study materials. Chandratre and Daroedono *et al.* argue that the situation of limited access to education and updated study materials forced lecturers and students to adjust their teaching and learning methods [4,6]. The COVID-19 pandemic pushed Indonesian higher education institutions to accelerate the use of digital technologies for learning, including those medical universities that might have resisted e-learning previously [7–9]. As in many other contexts, this shift to e-learning was not always successful due to a lack of infrastructure, staff capacity, student response, and limited internet access in some areas [7].

Despite these struggles, the sudden shift to distance e-learning clearly showed the potential benefits of e-learning and blended learning, such as the flexibility it gives to teaching and learning regarding time and space. The literature shows that it also enables self-directed learning and creativity in teaching methods [7,10]. Various tools and methods, such as adaptive tutorials, audiovisual clips, and interactive modules, can be used for e-learning, which can create more meaningful engagement between learners and instructors [10]. Online learning can enhance learning innovation in medical education [11] and potentially improve medical students’ higher-order thinking [12].

Blended learning, an approach that integrates online digital resources with crucial face-to-face interaction, offers a compelling, efficient, and cost-effective alternative to traditional classroom training, even for specialized areas such as medical statistics. This model has established a track record of consistent educational benefits within medical education; a recent systematic review confirmed that blended learning significantly enhances knowledge acquisition when compared to conventional pedagogical methods. Furthermore, the implementation of this instructional approach directly supports the Sustainable Development Goals (SDGs). Specifically, studying blended learning in tropical medicine contributes to SDG 3 (Good Health and Well-Being) by strengthening the health workforce’s capacity to effectively address tropical and neglected diseases. Simultaneously, it aligns with SDG 4 (Quality Education), as it promotes equitable, inclusive, and flexible access to essential medical education. Given these academic benefits and global development goals, the investigation of blended learning within the context of tropical medicine is both a strategic and necessary area of study [13].A significant obstacle to economic progress in Indonesia is the prevalence of Neglected Tropical Diseases (NTDs) among the country’s 111 million poorest people. These diseases perpetuate poverty and hinder the potential for economic growth in the region. Indonesia’s citizens face a significant burden of Neglected Tropical Diseases (NTDs). These diseases include worm infections like soil-transmitted helminths (STH) and lymphatic filariasis (LF), as well as bacterial infections like yaws and leptospirosis. Therefore, it is important for Indonesian medical students to master the management of tropical diseases.

Several quantitative studies have been conducted in Indonesia and elsewhere on using e-learning in education during the COVID-19 pandemic [5,7,14]. For example, Skendro et al. [15] investigated the use of e-learning in the context of sports sciences during COVID-19, and Jovanka et al. [14] conducted a quantitative study on the factors affecting the intention to use e-learning. Here, we present the findings of a mixed-methods study conducted in the aftermath of the COVID-19 pandemic, providing an in-depth understanding of the use of e-learning resources on tropical medicine in higher education institutions in Indonesia. This study contributes to the growing but still limited body of literature on e-learning resources for medical education in LMIC [1]. The research was conducted as part of a larger Erasmus+ capacity building project, ‘Health Information and Technology for Improved Health Education in South-East Asia’ (HITIHE), conducted in Indonesia and Cambodia.

## Methods

### Study setting

In 2022, there were a total of 92 medical schools in public or private universities in Indonesia. Most medical faculties divide their curriculum into modules based on organ systems. The curriculum includes specific abilities such as research, professional development, and procedural skills [15]. Early clinical exposure is achieved by using clinical scenarios to trigger discussion. The undergraduate medical program in Indonesia consists of two years of clinical work after at least three and a half years of preclinical study [16]. Meanwhile, the medical residency program takes four years of studying in clinical settings after graduating as a general physician. To ensure the quality of institutions that provide medical education and to ensure that doctors have comprehensive training, Indonesia has an independent accreditation institution called the Independent Accreditation Institute for Health Universities (Lembaga Akreditasi Mandiri Perguruan Tinggi Kesehatan/LAM-PTKES). The Indonesian accreditation standards are adapted from the World Federation of Medical Education (WFME). Before 2022, institutions were classified by the LAM-PTKES into three levels of accreditation: A, with A being the highest, and C being the lowest level of accreditation [17].

### Study design

This was a mixed-methods study that combined quantitative and qualitative research approaches. We conducted a survey that aimed to describe the pattern of e-learning use among medical students and a qualitative study among students and academic staff to understand the use of e-learning platforms on tropical medicine in medical schools in Indonesia. The data collection was conducted from July to September 2022. To improve the trustworthiness of the findings, we triangulated the survey findings and qualitative data during the analysis [18].

### The Quantitative Study

We employed stratified random sampling to select 13 medical faculties out of the total 92 faculties in Indonesia in 2022 based on the accreditation level by LAM-PTKes and regions designated by the Indonesian Association of Medical Education Institutions (AIPKI). These 13 medical faculties were chosen to ensure representation across all accreditation levels and AIPKI regions, thus providing a comprehensive overview of e-learning practices in tropical medicine across diverse institutional contexts in Indonesia. In total, five medical institutions with “A” accreditation, four medical institutions with “B” accreditation, and three medical institutions with “C” accreditation were selected. Then, we contacted academic staff and representative students in each selected institution, known as Persons in Charge (PICs). Each PIC selected study participants by convenience sampling, conducted informed consent, and aided the research team in communicating with agreed participants. The participants were invited by email or personal message using WhatsApp from a PIC of each institution. The minimal sample size for this study is 765 respondents, which was calculated based on a confidence level of 95%, and we got 888 students as the sample in this study.

**Fig 1:**
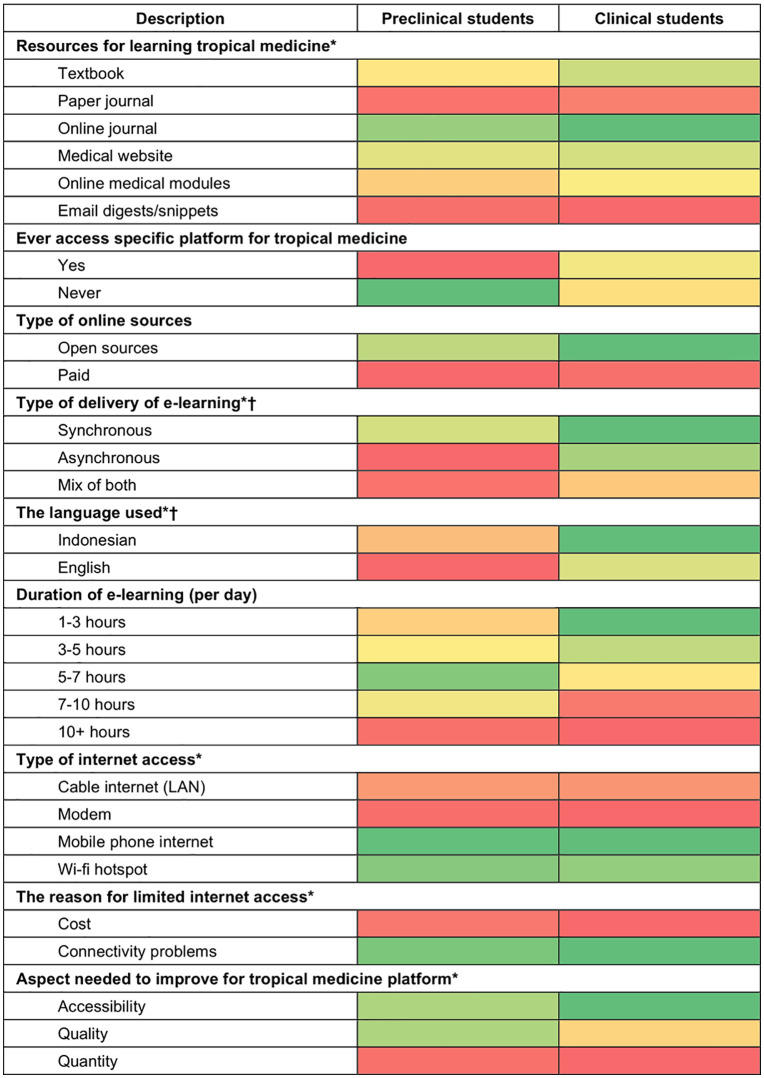
Preferences for learning method: comparisons by type of students.

**Fig 2:**
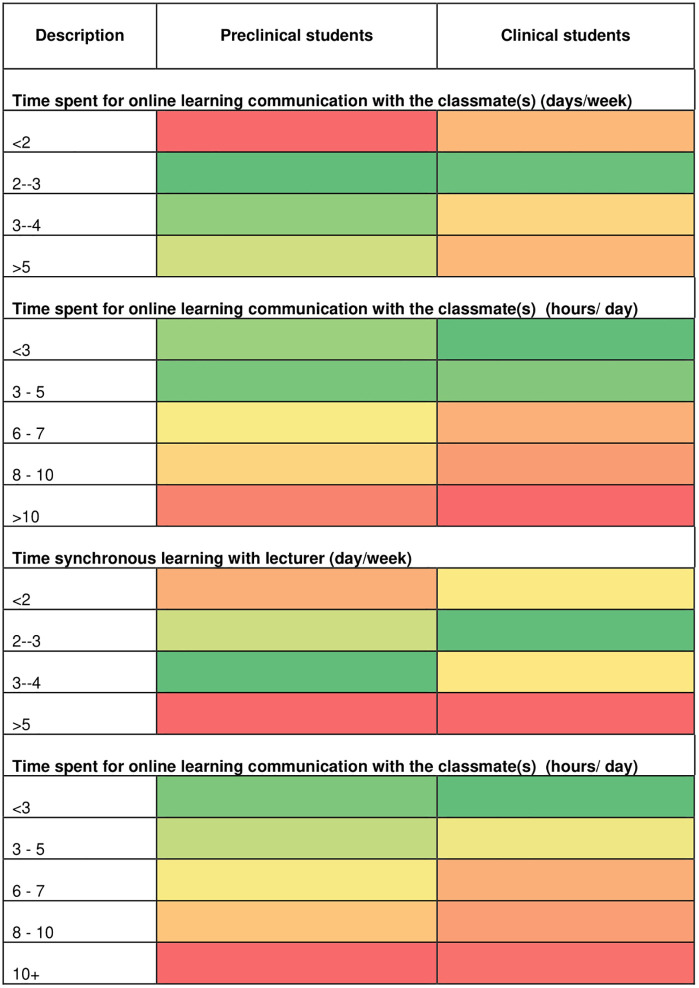
Time spent on e-learning platform based on student’s current study progress.

The data were collected using an online structured self-reported questionnaire in Bahasa Indonesia. The online questionnaire was sent to university PICs to distribute among the students. The questionnaire includes 14 questions on students’ experience with e-learning. The following topics were researched: (i) types of online learning, (ii) duration of e-learning, (iii) continuous medical education, (iv) use and ability of e-learning, in addition to (v) sociodemographic characteristics. A pilot study was conducted to ensure the validity and reliability of the questionnaire, resulting in 14 valid and reliable usage questions. Data were analysed using descriptive statistics, and comparisons between groups were conducted using Chi-squares. To further describe the preference and time spent on e-learning platform of preclinical and clinical students, we develop heatmap (Fig 1 and 2), with gradient of colours red showing less than 50% of students reported to be in the category, yellow showing approximately 50% of students reported to be in the category, and green showing more than 50% of students reported in the categories.

### The Qualitative Study

The data were collected through five online focus group discussions (FGDs). Informants were recruited using purposive sampling. Respondents from both preclinical and clinical medical students were selected based on specific criteria: they needed to be affiliated with the 13 institutions participating in the quantitative study, complete the quantitative survey questionnaire, and be selected by the PIC. Lecturer respondents were chosen based on criteria stipulating that they were either responsible for or involved in teaching the tropical infection block or were clinical rotation lecturers in the field of internal medicine. We distributed the invitation to all institutions involved, and only respondents who voluntarily wanted to come attended as study participants. The five online FGDs were conducted among 6–8 participants. The three FGDs were conducted among students: one preclinical students’ group, one clinical students’ group, and one group was a mix between preclinical and clinical students. Two other FGDs were conducted among lecturers from the 13 universities included in the studies. Five FGDs were conducted to obtain maximum variations of respondents, and were considered adequate to achieve data saturation.

Each target group (i.e., lecturers, clinical and preclinical students) was invited to a different FGD session, enabling an in-depth discussion according to the participant s’ background. This would also limit bias as students might provide different answers when their lecturers are present. FGD enriched the data by allowing different points of view to emerge in a discussion. The FGD guide consisted of questions regarding the learning management system from the university, websites visited for e-learning (i.e., YouTube, PubMed, Google Scholar), e-learning platforms on tropical medicine, time spent on e-learning, and expectations regarding e-learning. All FGDs were recorded, and notes were taken. The FGDs were transcribed independently by two researchers after each session.

Thematic analysis was used to analyse the data manually [19,20]. First, the interview data were read, reviewed, and coded, followed by grouping into categories based on patterns.

### Research ethics

The mixed-methods study was conducted according to the principles stated in the Declaration of Helsinki (Ethical Principles for Medical Research Involving Human Subjects) as amended in 2008. This study protocol was reviewed and approved in Indonesia by the Research Ethical Committee of the Faculty of Medicine Universitas Sebelas Maret, Indonesia (No: 42/UN27.06.11/KEP/EC/2022) and in Belgium by the Institutional Review Board of the Institute of Tropical Medicine in Antwerp (ITM IRB, n. 1580/22 “Mapping, characterization and perceptions of the current offer of online information and e-learning on tropical medicine in Cambodia and Indonesia: a mixed-method study; v. 2.0, dd 15/06/2022). Written consent was obtained from the participants for the quantitative and qualitative study.

## Results

### Study participants

We collected and analysed the questionnaire from a total of 888 medical students, consisting of preclinical (i.e., undergraduate students without clinical experience, 57.0%, n = 506) and clinical students (i.e., undergraduate students who are doing 2 years of clinical work, 43.0%, n = 382). The mean age for preclinical students is 21 years old, while for clinical students is 23 years old. Females dominated the population both in preclinical (67.2%, n = 340) and clinical students (71.5%, n = 273). The data showed a similar distribution of institutions’ accreditations both in preclinical and clinical groups, dominated by institutions with accreditation A (37.2%, n = 330), followed by accreditation B (35.4%, n 314) and C (27.4%, n = 244), respectively. Lastly, based on the type of university, there were more preclinical students coming from private universities (60.7%, n = 307). Meanwhile, clinical students were distributed more evenly between private universities (49.2%, n = 188) and public universities (50.8%, n = 194). There were no statistical differences based on the sociodemographic characteristics of the students (Table 1).

**Table 1:** The characteristics of study participants (students) in the survey (quantitative study).

Description	Preclinical students (%) n = 506	Clinical students (%) n = 382	Total n = 888
**Age**			
Mean±SD	20.8 ± 1.136	22.5 ± 1.207	21.5 ± 1.439
**Gender**			
Male	166 (32.8)	109 (28.5)	275 (31.0)
Female	340 (67.2)	273 (71.5)	613 (69.0)
**University’s accreditation**			
A	184 (36.4)	146 (38.2)	330 (37.2)
B	180 (35.6)	134 (35.1)	314 (35.4)
C	142 (28.0)	102 (26.7)	244 (27.4)
**University’s type**			
Public university	199 (39.3)	194 (50.8)	393 (44.3)
Private university	307 (60.7)	188 (49.2)	495 (55.7)
**Total**	**888 (100)**		

For the qualitative study, 12 lecturers (seven females, five males), eight preclinical students (six females, two males), and 11 clinical students (nine females, two males) from 11 medicine schools from several islands in Indonesia participated in the FGDs. Seven female and five male lecturers from eleven of the 13 institutions participated.

### Resources and accessibility of tropical medicine e-learning

In Indonesia, users can access several resources to learn about tropical medicine. In this study, the three most used are online journals (65.3%, n = 580), medical websites (40.1%, n = 356), and textbooks (35.8%, n = 318), followed by online modules, paper-based/printed journals, and newsletters sent by email. In general, a higher proportion of clinical students access the resources of tropical medicine e-learning compared to preclinical students. The most frequently accessed e-learning resources accessed by clinical students were online journals (74.1%, n = 283), followed by textbooks (45.0%, n = 172). Meanwhile, preclinical students also frequently access online journals (58.7%, n = 297), followed by medical websites (38.1%, n = 193). There were significant differences in the use of textbooks (p-value <0.001), online journals (p-value <0.001), and online medical modules (p-value = 0.018) between the groups. These findings are further depicted in Fig 1, showing heatmap of preferences of learning methods, comparing the clinical and preclinical students. The red colours indicate lack of preferences by medical students. For both clinical and preclinical students, there were lack of preferences in accessing paper journal and email digest for resources.

Our findings from the FGD, Google Scholar was the first access point for students to acquire scientific information on tropical medicine. They also find information via Medscape (i.e., an online tool for health workers), Osmosis (i.e., an all-in-one health education platform), and the MSD Manual (The Merck Manuals containing medical references). They found helpful sources with problem trees/disease charts, such as the Calgary Guide. Students identified the following international journals containing interesting information about tropical diseases: PubMed, NCBI, Elsevier, and CDC. However, medical students commonly use social media, such as YouTube, as another important source.

“*E-learning access via the web, of course, is more often via the web, doc. So, for e-learning, it is mostly via YouTube, so it is like videos because it is more communicative too, like usually videos from Armando Hasudungan* [Armando Hasudungan is a medical content creator who posts educational videos on YouTube]*. Then, for other media, usually from several institutions that facilitate our study of medicine like that, doc. …. And besides that, usually, for example, via Instagram, like social media often shares online quizzes, like that, mostly, I learn from there, doc.*” (Student 14, female)

Preclinical students mostly never accessed a specific e-learning platform for tropical medicine (68.2%, n = 345); meanwhile, the majority of clinical students accessed specific e-learning platforms for tropical medicine (51.6%, n = 197). This finding showed statistically significant differences (*p*-value of <0.001) in both groups ever accessing e-learning platforms for tropical medicine. Almost all online sources were open-source (98.3%, n = 352), with users accessing without a subscription from their institution or out-of-pocket payment. A significant difference was also found in the use of open-source access between preclinical students and clinical students (*p*-value of <0.001). Clinical students used open-source access more often (50.8%, n = 194) than preclinical students (31.2%, n = 158).

The quantitative data study showed a significant difference in methods of e-learning access (*p*-value = 0.05). Synchronous e-learning was the most used method (45.3%, n = 162), followed by asynchronous e-learning (29.9%, n = 107) and a combination of both (24.9%, n = 89). The languages, Indonesian and English, used on the tropical medicine platform also showed a significant difference (*p*-value of <0.001) across both groups, although Indonesian was more used in tropical medicine platforms in preclinical group (26.1%, n = 132) and clinical group (44.8%, n = 171). The different preferences of preclinical and clinical students are further depicted in Fig 1, in which the heatmap showed red colour (low proportion) of preclinical students in accessing asynchronous or mix of both synchronous and asynchronous type of e-learning platform as well as English resources, whereas more clinical students accessed them (green colour).

Clinical students accessed specific platforms for tropical medicine more frequently than preclinical students, with an average access time of 1–3 hours (40.3%, n = 154). Preclinical students showed a preference for synchronous platforms over asynchronous (23.6%, n = 38) and mixed types (25.5%, n = 41), accessing the platform with an average daily usage of 5–7 hours (35.8%, n = 181). These findings suggest different patterns, which was that preclinical students were more likely to use a mix of both synchronous and asynchronous types of learning, while clinical students were more likely to use asynchronous. Preclinical students showed a longer duration of e-learning access (Table 2).

**Table 2:** Preferences for learning method: comparisons by type of students.

Variable	Preclinical students (n total = 506)	Clinical students n total = 382	Total	*p-*value
**SOURCES OF INFORMATION ON TROPICAL MEDICINE**				
Resource about tropical medicine*				
Textbook	146 (28.9)	172 (45.0)	318 (35.8)	<0.001***
Paper journal	22 (4.3)	26 (6.8)	48 (5.4)	0.109
Online journal	297 (58.7)	283 (74.1)	580 (65.3)	<0.001***
Medical website	193 (38.1)	160 (41.9)	353 (39.8)	0.259
Online medical modules	119 (23.5)	117 (30.6)	236 (26.6)	0.018*
Email digests/snippets	9 (1.8)	7 (1.8)	16 (1.8)	0.952
**Access specific platform for tropical medicine**				
Yes	161 (31.8)	197 (51.6)	358 (40.3)	<0.001***
Never	345 (68.2)	185 (48.4)	530 (59.7)	
**Type of online sources†**				
Open sources	158 (31.2)	194 (50.8)	352 (39.6)	<0.001***
Paid	12 (2.4)	13 (3.4)	25 (2.8)	0.358
**Type of delivery of e-learning†**				
Synchronous	82 (50.9)	80 (40.6)	162 (45.3)	0.05*
Asynchronous	38 (23.6)	69 (35.0)	107 (29.9)	
Mix of both	41 (25.5)	48 (24.4)	89 (24.9)	
**USAGE OF PLATFORM/COURSES ON TROPICAL MEDICINE**				
**The language used†**				
Indonesian	132 (26.1)	171 (44.8)	303 (34.1)	<0.001***
English	98 (19.4)	126 (33.0)	224 (25.2)	<0.001***
**Duration of e-learning on tropical medicine (per day)**				
1 - 3 hours	84 (16.6)	154 (40.3)	238 (26.8)	<0.001***
3 - 5 hours	100 (19.8)	106 (27.7)	206 (23.2)	
5 - 7 hours	181 (35.8)	73 (19.1)	254 (28.6)	
7 - 10 hours	108 (21.3)	28 (7.3)	136 (15.3)	
10+ hours	33 (6.5)	21 (5.5)	54 (6.1)	
**Type of internet access**				
Cable internet (LAN)	100 (19.8)	70 (18.3)	170 (19.1)	0.590
Modem	27 (5.3)	14 (3.7)	41 (4.6)	0.240
Mobile phone internet	415 (82.0)	315 (82.5)	730 (82.2)	0.864
Wi-fi hotspot	374 (73.9)	270 (70.7)	644 (72.5)	0.285
**The reason for limited internet access**				
Cost	194 (38.3)	138 (36.1)	332 (37.4)	0.500
Connectivity problems	358 (70.8)	282 (73.8)	640 (72.1)	0.313
Aspect needed to improve for tropical medicine platform*				
Accessibility	409 (80.8)	323 (84.6)	732 (82.4)	0.149
Quality of platform	409 (80.8)	279 (73.0)	688 (77.5)	0.006**
Quantity	281 (55.5)	206 (53.9)	487 (54.8)	0.634
**ACCESS/PERSONAL DEVICE**				
**Personal device ownership**				
Yes	494 (97.6)	376 (98.4)	870 (98.0)	0.619
Yes, but it doesn’t work well	11 (2.2)	5 (1.3)	16 (1.8)	
No (sharing with others)	1 (0.2)	1 (0.3)	2 (0.2)	
The device used to access online information*				
Laptop	432 (85.4)	294 (77.0)	726 (81.8)	0.001***
Desktop computer	12 (2.4)	13 (3.4)	25 (2.8)	0.358
Tablet	101 (20.0)	85 (22.3)	186 (20.9)	0.406
Smartphone	448 (88.5)	340 (89.0)	788 (88.7)	0.827

From the lectures’ point of view, qualitative data showed that after COVID-19 most lecturers aimed to have blended learning or hybrid classes as they found it essential to return to the in-person learning method. Lecturers were motivated to engage in e-learning as they found that students needed to develop digital skills and perceived that e-learning helps to achieve learning objectives. A regularly mentioned benefit of e-learning was the ability to keep objective records of students’ learning activities, such as frequency of access and duration of access. They also mentioned some negative aspects of e-learning tools, including the limited possibility of interacting with students and technical problems.

“*In the future, do we choose offline or online? In my opinion, both are good. Tailored to the needs, the effectiveness of the process of what we do. So, in the future, in my opinion, it will not stop: e-learning will be even more effective, even better than what is currently running. That is all with the constraints earlier that had to be improved; the admin and the server were also repaired. And that, in my personal opinion, is related to choosing between online learning or face-to-face learning. If it is for theory classes, I think it is better if you start from home because you can be on time while the next lecture must also be on time. So, the schedule is on time; for learning clinical cases, it has to be done in a practical manner*.” (Lecturer 1, female)

“*For us, we will continue to be more face-to-face even though we will still encourage online learning so that it will be more blended.*” (Lecturer 8, male)

*“Yes, well, for me, using Google Classroom in our learning activities has helped me achieve our learning goals. So, we think it is already good, but there are still some aspects that we might need to improve, especially the interaction between students and lecturers, which is still minimal. There are technical constraints from the lecturer. Even though we are also taking precautions, such as having an admin who is in charge of uploading lecturer materials and also responding to feedback, on a per-assembly basis, we have the admin use Google Classroom. It was like that, but thank God the learning objectives were achieved, and we can also monitor who is late, who is early, or who does not complete their assignments, and who is active in discussions, so for me, it is in accordance with achieving our learning goals. That is quite effective, in my opinion.” (Lecturer 4, male)*

Most students had the same vision as the lecturers and preferred blended learning. Students found that e-learning activities such as tutorials are enjoyable to engage with. E-learning was perceived to be quite effective because the sources were more updated and accessible, and the atmosphere was more conducive and more self-focused. Some students also found it easier to read slides and tutorial notes using their laptop screen than the classroom presentation. E-learning was found to be well-suited for theoretical classes, class discussions, and collecting assignments and exams. Students stressed that learning skills required offline teaching in hospitals and during skills laboratories, practical sessions, and community service. This was a recurring topic during focus group discussions, with students agreeing that online skills learning during COVID-19 was ineffective. During COVID-19, students were expected to make videos of their skills and upload them on a platform to be reviewed by lecturers. Students reported that applying theory related to practical skills was difficult without direct training. They also found that online skills learning was ineffective due to the lack of user-friendliness of the platform interface and the limited direct feedback by teachers on their development. Infrastructural aspects, such as limited internet access, further inhibited online skills learning, whereby students living in places with limited internet connectivity were disadvantaged (e.g., students living in rural places with limited internet access). In addition to the previously mentioned factors, qualitative data showed that student learning motivation was important in understanding the effectiveness of online skills learning.

“*From me, if the skills lab were online, it would be much less effective, doctor. For example, if there were a skills lab as well, especially regarding tropical infections or tropical medicine, we should interact more with the community and so on. However, for me, tutorials were very effective if we used online learning. Because in the first place, we can learn more if there are more available sources. The online ones were more up-to-date, new, and also usually more complete than the printed sources or the sources in the offline mode*.” (Student 7, female)

Some students thought that overall e-learning was better and mentioned that the schedule for online discussions was more flexible than offline. Therefore, online discussions save time and costs. These students found that online tutorials and lectures made taking notes more enjoyable and efficient. They also found it beneficial to access the tutorials and lectures’ recordings and review them afterward. The students also seemed more active and engaged because of the available raise-hand feature. The online assignment was found to be more environmentally friendly because it was paperless. However, these students also reported many infrastructural obstacles, such as unstable internet connections.

The students who preferred offline learning argued that the material presented in a traditional classroom setting was easier to understand. They found that offline tutorials were livelier, sharpened their knowledge, and allowed them to practice communication skills with patients without reading references. The students felt that in face-to-face classes, the lecturer took their role as a tutor more explicitly, allowing them to learn more efficiently.

### Access to the internet

To access online resources used for education, participants reported using mobile phone internet (82.2%, n = 730) and WI-FI hotspots (72.8%, n = 646) without any significant differences between preclinical and clinical students (Table 2). Other options were used occasionally, such as cable internet (19.1%, n = 170) and modem (4.6%, n = 41).

Accessing e-learning resources came with several challenges, including connectivity problems (72.1%, n = 640) and the associated cost of internet access (37.4%, n = 332). Only a few students (9%, n = 80) reported having no access problems. Most students had a well-working personal device(s) (98.0; n = 870). The most widely used types of devices were smartphones (88.7%, n = 788), utilized by both preclinical students (82.0%, n = 415) and clinical students (82.5%, n = 315). Additionally, there were significant differences (*p*-value = 0.001) in the use of laptops (81.8%, n = 726) between preclinical students (85.4%, n = 432) and clinical students (77.0%, n = 294) (Table 2).

### Aspects needed to improve on tropical medicine e-learning

Medical students mentioned that current international and local platforms containing information about tropical medicine should become more accessible (82.4%, n = 732; preclinical group 80.8%, n = 409; clinical group 84.6%, n = 323). However, there was a significant difference in the need to improve the quality of the platform (*p*-value of 0.006) between the preclinical group (80.8%, n = 409) and clinical group (73.0%, n = 279). The aspect least needed to improve is the quantity of tropical medicine platforms, both in the preclinical group (55.5%, n = 281) and clinical students (53.9%, n = 206) (Table 2). The students perceived these international platforms as easy to read and engaging with several features, such as videos and animations. These features were comprehensive and well-structured, enabling them to discuss the pathophysiology and management of tropical diseases, and they were able to develop clinical reasoning. Several drawbacks of these international e-learning platforms were (i) the difficulty of registration, (ii) irrelevant content on tropical medicine, and (iii) a lack of updates.

“*Perhaps for the platforms outside of campus, there were medical videos, especially for tropical infections. I think it is more relevant to watch videos from Indonesia because tropical infections in Indonesia are different from tropical infections outside Indonesia. So, it is more in-depth, and the guidelines were also more relevant to the situation here. Then, for the drawbacks, maybe it is less attractive in terms of pictures. However, in terms of understanding or material, it is much better if (we) look at the sources from Indonesia, doc*.” (Student 1, female)

Medical students also had access to various local (Indonesian) platforms that provided educational information, one of which included tropical medicine. These e-learning platforms included local medical videos, Indonesian-language journals specifically for infectious diseases, consensus information from various medical associations (i.e., Internal Medicine Associations, Infectious Disease Associations, etc.), and several independent medical e-learning institutes such as Interna is Fun, Dopamed.id, Medsense, Mediko, Sejawat.idn, and Asclepio. There were also local applications like M3 Kedokteran Umum that contained books, journals, and guidelines.

In general, local websites were of good quality and had some promising applications. Students found them easy to understand since they contained structured materials that included relevant information about epidemiology and the management of tropical diseases and medicine. However, the visuals were criticized as unattractive. These findings were relevant to quantitative data, indicating the need for improvement in the quality of e-learning platforms.

Access to the most recent local journals was needed since tropical medicine cases vary based on geography, and the management of these cases can differ from one source to another. Also, the theories presented by lecturers may differ from those in local journals. Despite the prevalence of these local platforms, international platforms were accessed more often by medical students because they were perceived to be more helpful for learning. International platforms were considered to be more up-to-date, interactive, and engaging.

Another aspect to improve is the user interface of e-learning platforms. The respondents expressed their desire for an attractive, interactive tropical medicine e-learning platform with more comprehensive and relevant materials. Clinical students specifically mentioned the need for open access to local journals or repositories, particularly those related to tropical medicine. They suggested that information about these journals and repositories should be integrated into a single website and organized by theme. Other beneficial features include easy registration, an attractive layout, tools to enhance clinical research, and interactive features such as quizzes, open discussion forums, games, and videos.

“*(It is hoped) that the learning platform could be categorized, for example, tropical medicine and internal medicine, those will help us to access the journal, and if it can, it is better if there is also a website with quizzes, if we only read texts from the platform, it would be boring, so with quizzes, hopefully, it would be more interesting. So that after the reading, there would be a quiz that tests our memory (and understanding) of the material.*” (Student 16, female)

### Students’ duration of e-learning access

E-learning was primarily delivered synchronously (45.3%, n = 162), but many respondents also engaged in asynchronous e-learning or a combination of both (Table 3). To provide a clearer description, we also include graphs [S1, S2, S3, and S4 Fig] in the supporting information. Students were often actively engaged with peers about the medical information they found online. Students often spent multiple days per week communicating about e-learning (5 days per week, 21.7%, n = 193). However, many students also spent 2 (18%, n = 160) or 3 days (19.49%, n = 172) per week) on communication about online learning per week. During those days, most students spent less than five hours daily, i.e., (3–5 hours: 39.6%, n = 352; < 3 hours: 38.4%, n = 341). Synchronous learning occurred five days per week (24.8%, n = 220) but often less than three hours per day (54.4%, n = 483). This indicates that e-learning and blended learning are still common ways to teach medicine in Indonesia, even after COVID-19. Most students mentioned they spent 5–7 hours per/day aiming to identify information on tropical medicine on general websites where they find information on medicine. The different pattern in time spent on e-learning platform by preclinical and clinical student is further describe in the heatmap (Fig 2).

**Table 3:** The Interactivity of the Online Learning Process.

Variable	Preclinical students (n, %)	Clinical students (n, %)	Total	*p-*value
**Time spent for online learning communication with the classmate(s) (days/week)**				
< 2	34 (6.7)	69 (18.1)	103 (11.6)	<0.001***
2 - 3	173 (34.2)	159 (41.6)	332 (37.4)	
4 - 5	159 (31.4)	85 (22.3)	244 (27.5)	
> 5	140 (27.7)	69 (18.1)	209 (23.5)	
**Time spent for online learning communication with the classmate(s) (hours/ day)**				
< 3	167 (33.0)	174 (45.5)	341 (38.4)	0.001***
3 - 5	206 (40.7)	146 (38.2)	352 (39.6)	
6 - 7	62 (12.3)	28 (7.3)	90 (10.1)	
8 - 10	48 (9.5)	23 (6.0)	71 (8.0)	
> 10	23 (4.5)	11 (2.9)	34 (3.8)	
**Time synchronous learning with lecturer (days/ week)**				
< 2	101 (20.0)	95 (24.9)	196 (22.1)	<0.001***
2 - 3	142 (28.1)	158 (41.4)	300 (33.8)	
4 - 5	187 (37.0)	92 (24.1)	279 (31.4)	
> 5	76 (15.0)	37 (9.7)	113 (12.7)	
**Time synchronous learning with lecturer (hours/ day)**				
< 3	256 (50.6)	227 (59.4)	483 (54.4)	0.001***
3 - 5	146 (28.9)	111 (29.1)	257 (28.9)	
6 - 7	64 (12.6)	22 (5.8)	86 (9.7)	
8 - 10	37 (7.3)	17 (4.5)	54 (6.1)	
> 10	3 (0.6)	5 (1.3)	8 (0.9)	

### Learning management system

FGD with lecturers indicated that each university has a Learning Management System (LMS). Some medical universities already had an LMS before COVID-19; however, the LMS was increasingly used in those cases, and lecturers discovered new features during the pandemic. Students and lecturers knew the LMS offered features that included sharing lecture sources and modules, e-books and journal links, tutorials and discussions, assignment collecting, evaluations, quizzes, exams, and student grades. A few universities have already used e-learning platforms for midterm and final-term exams. In contrast, the rest still used it only for computer-based tests (CBT) for multiple choice questions (MCQ) and final block exams. The majority of universities with A-level accreditation mainly use the LMS for midterm and final exams. Preclinical students were more likely than clinical students to use the LMS platform. Some of the existing LMS had technical issues, while others already had a designated team to prevent and tackle technical issues, such as network issues.

*“...E-learning is quite user-friendly too; in the beginning, it needed many revisions, but in hindsight, it has gotten better. However, what was often an obstacle may be the network.”* (Lecturer 5, female)

Students found the user interface of the learning platform not always interesting because of the amount of text and the lack of highlights about which information is most relevant. In particular, clinical students mentioned that the LMS had missed some information and that the learning sources were absent. The lecturers mentioned that some lecturers needed to familiarize themselves with the LMS and were using the platform irregularly. The qualitative research indicated that lecturers and students need to be made aware of possible regulations regarding the use of LMS in the universities included in the qualitative research.

*“..... Yes, we have one [i.e., LMS]); maybe it can be called an e-learning platform. We have something like that, so it is a lecture-containing blog or clinical skills learning [CSL] blog. It has a blog called Lentera, and the lecturers upload their lectures that students can access, for example, slides from the lecturer.*” (Lecturer 1, female)

Some universities provided online libraries for eBooks, journal repositories, online courses, e-learning websites, and a subscription or access to international journals on tropical medicine (such as Elsevier and CDC). Only after the pandemic did several universities begin improving and using social media (YouTube) and external websites such as Google Scholar and PubMed for daily teaching processes.

### Motivation to use e-learning platforms

Students utilize international and local e-learning platforms with various objectives during their studies. Firstly, they utilize the platforms to complete their assignments and prepare for exams. Secondly, they aim to improve their clinical skills by watching videos and reading about clinical knowledge online. Thirdly, the platform material serves as a basis for discussions with friends in informal settings and lecturers in class settings. Moreover, students also use the information to prepare for their clinical rotation activities. Lastly, these platforms are a common source of information, enabling them to study the most common diseases and compare them with the patients they are meeting, support clinical knowledge when meeting patients, and learn about new conditions and engaging specialties.

Qualitative research showed the perceived benefits of an online platform on tropical medicine to support clinical students who are future professionals in their practice. Therefore, students must have digital skills to update their knowledge throughout their careers.

*“I use it mainly to do the task, as well as if I get a case when I am on a shift like directly searching ‘what were the symptoms, what was the therapy?’, so when we start taking the patient’s history, it is more directed.”* (Student 10, female)

*“In our infection block/module, the tutor (in the tutorial) required us to learn from journals and other sources, mainly for tutorial sessions. Another thing, when we sometimes do social services* (to citizens)*, we prepare materials about tropical infection, et cetera; other than that, maybe it can also be shared.“* (Student 3, female)

## Discussion and conclusion

In many LMIC, tropical infectious diseases have a significant impact if they are not treated immediately. Research has shown that medical students need to enhance their knowledge about tropical diseases by improving learning strategies, gaining digital skills, and using technological advances in learning [21]. This mapping study, conducted in the aftermath of the COVID-19 pandemic, explored the experience of medical students and lecturers with e-learning on tropical medicine in Indonesia. Based on our quantitative and qualitative analysis, we found that students and lecturers do not have access to a specific platform on tropical medicine and, therefore, retrieve information on tropical medicine from general peer-reviewed and non-peer-reviewed websites.

Pre-clinical and clinical students both had the main preference for online journals. Another study in Saudi Arabia found that students used online journals as a learning resource preference more than other options; they also were more likely to pass all modules [22]. However, there were significant differences between pre-clinical and clinical students in accessing online journals. The percentage of clinical students accessing online journals was greater than pre-clinical students. A study in Pakistan found that preclinical students had a negative perception of e-learning for several reasons, for example, connectivity issues, electricity issues, and less interaction with colleagues and teachers [23]. Another study in Korea compared pre-medical students in year 1, pre-medical students in year 2, medical students in year 1, and medical students in year 2, and found that medical students in year 2 scored the highest in self-directed learning skills in an online learning context [24]. Other studies in Nigeria indicated, although not statistically significant, that older medical students showed slightly higher satisfaction with online learning than younger students [25].

Students spent substantial time looking at general international and local platforms to retrieve information on tropical medicine. A qualitative study involving seven public and private allopathic US Medical Schools found that third-party learning resources (learning resources not created by medical schools) provided some benefits for learning [26].

Quantitative data showed a significant number of preclinical students accessed online journals (58.7%, n = 297), followed by medical websites (38.1%, n = 193), while clinical students mostly accessed e-learning platforms through online journals (74.1%, n = 283), followed by textbooks (45.0%, n = 172). However, our qualitative data shows that social media (YouTube) is also an important information source. Similar findings are also found in research from India that showed that social media is a source of information for medical students [27]. Indian medical students describe social media as easy to use, and it is perceived to impact their academic performance positively. However, using social media as a resource is also a concern, as information is only sometimes peer-reviewed or verified. Generally, the respondents hoped for an attractive and interactive e-learning platform with more comprehensive and relevant materials on tropical medicine for Indonesia. Based on this data, we argue that an open-access e-learning platform containing information on tropical diseases can be an interesting tool for students, lecturers, and healthcare professionals. High-quality and updated tropical medical and health science education content is needed to enhance student capacity and inform healthcare professionals. Educational material that considers the local context is lacking on international platforms. Students mentioned the relevance of interactive clinical cases from Indonesia, which could help them learn about tropical medicine. Interactive clinical cases can also motivate students and be helpful when offline learning interaction returns to being limited [28] or when it concerns rare medical conditions. A previous study found that interactive clinical cases could help undergraduate students learn about antibiotic choices despite not meeting patients directly [29]. Research from India showed that interactive clinical cases could support students’ self-directed learning when thoughtfully integrated in blended learning formats [30].

Our study found that 98% of Indonesian medical students have personal devices, but internet access is still a barrier. This confirms research from some other LMIC countries, clarifying that poor internet coverage and financial constraints to use the internet are still considerations when investing in e-learning [5,31]. Furthermore, our study found that 98% of medical students choose open resources due to financial constraints. To answer those problems, universities with medical faculties should subscribe to journals as e-learning resources for students and provide adequate infrastructure to facilitate e-learning, particularly internet connection [5,32]. Lecturers also need support from techno-pedagogical staff and IT services to improve their capability to provide e-learning. Students and lecturers agree that faculties should take responsibility for building and maintaining infrastructure [33]. Despite the existing structural barriers to e-learning in medical universities in Indonesia, the general attitude of students and lecturers towards blended learning was positive. Distance e-learning was implemented during the COVID-19 pandemic, and many medical students and lecturers experienced the benefits. The perceived advantages of e-learning are the increased opportunities for social interaction and communication with peers and lecturers, independent of place and time.

Students and lecturers found that, as future physicians, medical students must acquire digital skills to quickly retrieve the latest medical information and continue their professional education [34]. This skill is particularly important as medical technology and knowledge are rapidly evolving. In this study, most medical students and lecturers preferred blended learning. They were critically aware that various learning objectives require different teaching activities. Similar to medical faculties in Saudi Arabia, students and lecturers mentioned that theoretical content could be delivered online, but offline learning is preferable for other domains, particularly clinical skills [35]. This study showed a general agreement among students and lecturers that learning clinical skills should happen in practice with patients, for example, when carrying out clinical learning in community settings.

This study also gave lessons that in the context of current medical students who are familiar with digital technology and also in the digital health system era, medical schools should consider implementing student-driven learning with advanced technology through self-learning and peer-studying while considering resource accessibility.

This study contributes to the current literature, with only a limited publication regarding tropical medicine e-learning platforms available from 2022−2025. For example, there was a previous study by Gaudin *et al*. comparing learning methods between e-learning and face-to-face to learn Lyme disease. The study found that e-learning was more effective than face-to-face learning based on pre-test and post-test scores [36]. For Indonesia, no similar study has been found on e-learning platforms for tropical medicine, especially in the post-COVID-19 period, when a major change happened to the teaching and learning process. Next, we have not found any studies discussing the availability of tropical medicine e-learning platforms and policy and curriculum in medical education in Indonesia. Hence, this paper puts its novelty and importance in this topic.

Despite its contribution, this study has several limitations: First, the survey sampling methods which might introduce bias. We used convenience sampling to select individual participants from each medical faculty member who might have tended to select students who actively engaged with the learning process, hence, prone to bias. Second, the limited generalizability of the study. While this study represents all regional areas and accreditation levels of medical schools, the number of participating medical schools and students/faculty were limited. Finally, the time gap between data collection and publication is relatively long, and changes may have occurred during this period. However, we believe that the findings provide important information that needs to be disseminated through this publication. In conclusion, the access and utilization of e-learning platforms on tropical medicine in Indonesia still need to be improved in many aspects, including the accessibility and quality of e-learning platforms. There was a higher proportion of clinical students accessing resources for e-learning in tropical medicine. However, there were similarities in the types of e-learning platforms accessed by pre-clinical and clinical students; for example, resources came mostly from online journals, textbooks, and medical websites. More students in both groups also opted for open sources more than paid platforms, used Indonesian as the primary language, and accessed using mobile phone internet and WI-FI hotspots. Several differences were also found between groups, where preclinical students mostly never accessed the e-learning platform of tropical medicine. In contrast, most clinical students and the pre-clinical group accessed the platform longer than the clinical group.

This study highlighted the need to optimize and adjust the access and utilization of tropical medicine e-learning platforms in Indonesia based on students’ level of study. Moreover, there is a growing need for accessible online learning platforms focused on tropical medicine to enhance medical education.

## Supporting information

S1 Fig Time spent on online learning communication with the classmate(s) (days/week). (TIFF)

S2 Fig Time spent for online learning communication with the classmate(s) (hours/day). (TIFF)

S3 Fig Time synchronous learning with lecturer (days/week). (TIFF)

S4 Fig Time synchronous learning with lecturer (hours/ day). (TIFF)
