# Supplementary figures and images for: Access and utilization of e-learning on tropical medicine at higher education institutions in Indonesia: A mixed-methods study
Source: PLoS One. 2025 Dec 31;20(12):e0335664. doi: 10.1371/journal.pone.0335664 (PMC12755740; doi:10.1371/journal.pone.0335664)

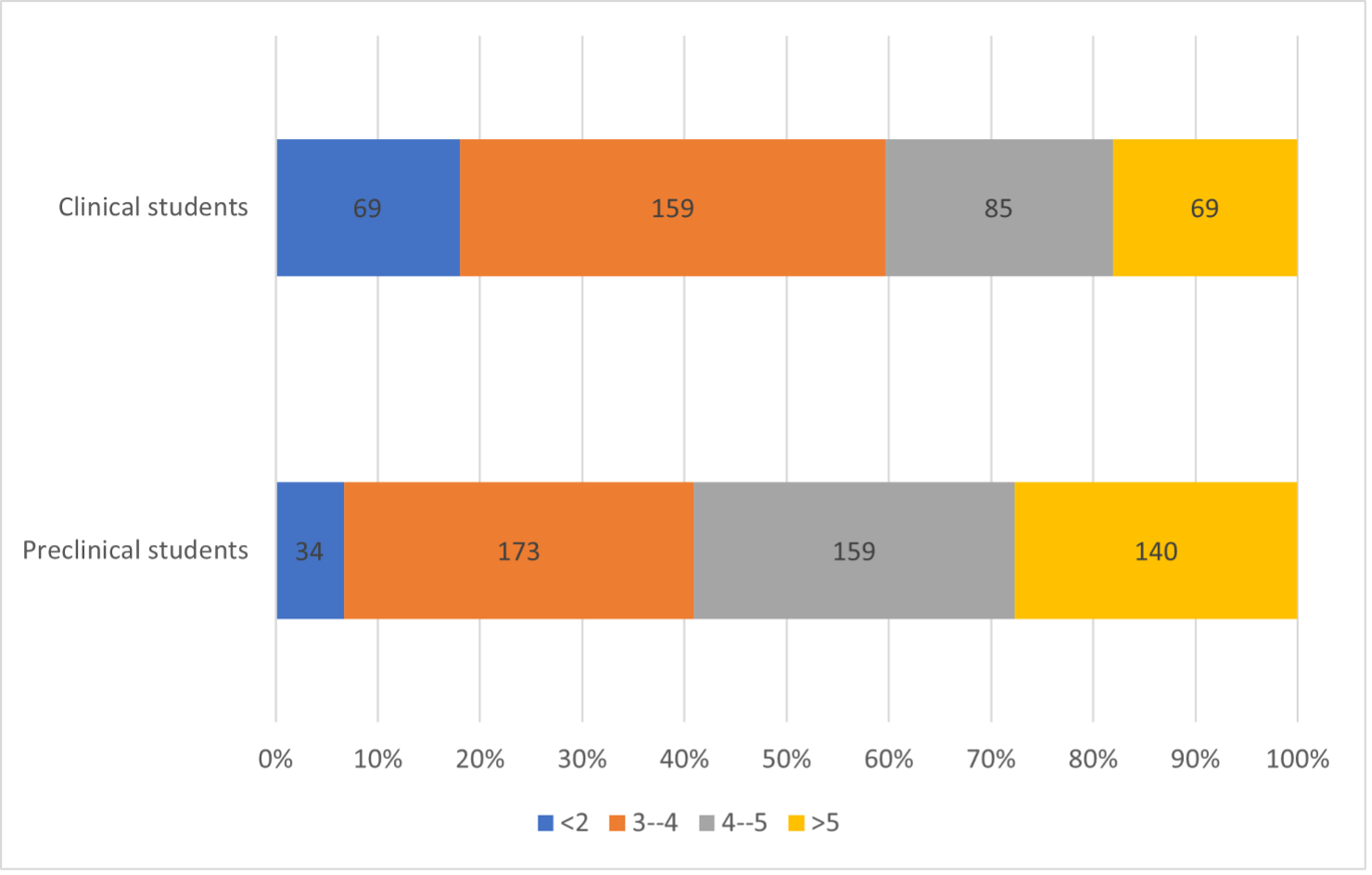

Supplement: S1 Fig — (TIFF) [file pone.0335664.s001.tiff]

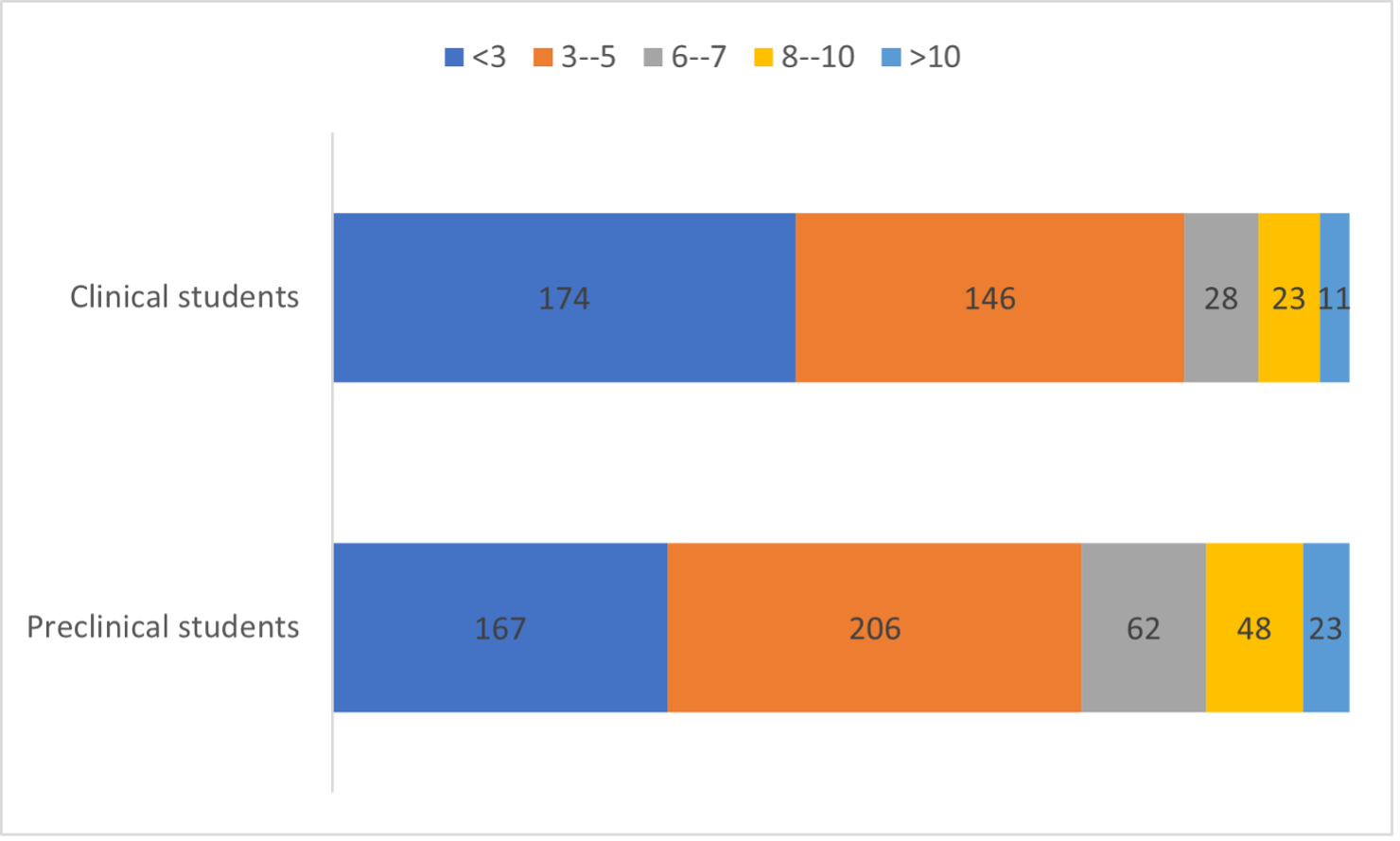

Supplement: S2 Fig — (TIFF) [file pone.0335664.s002.tiff]

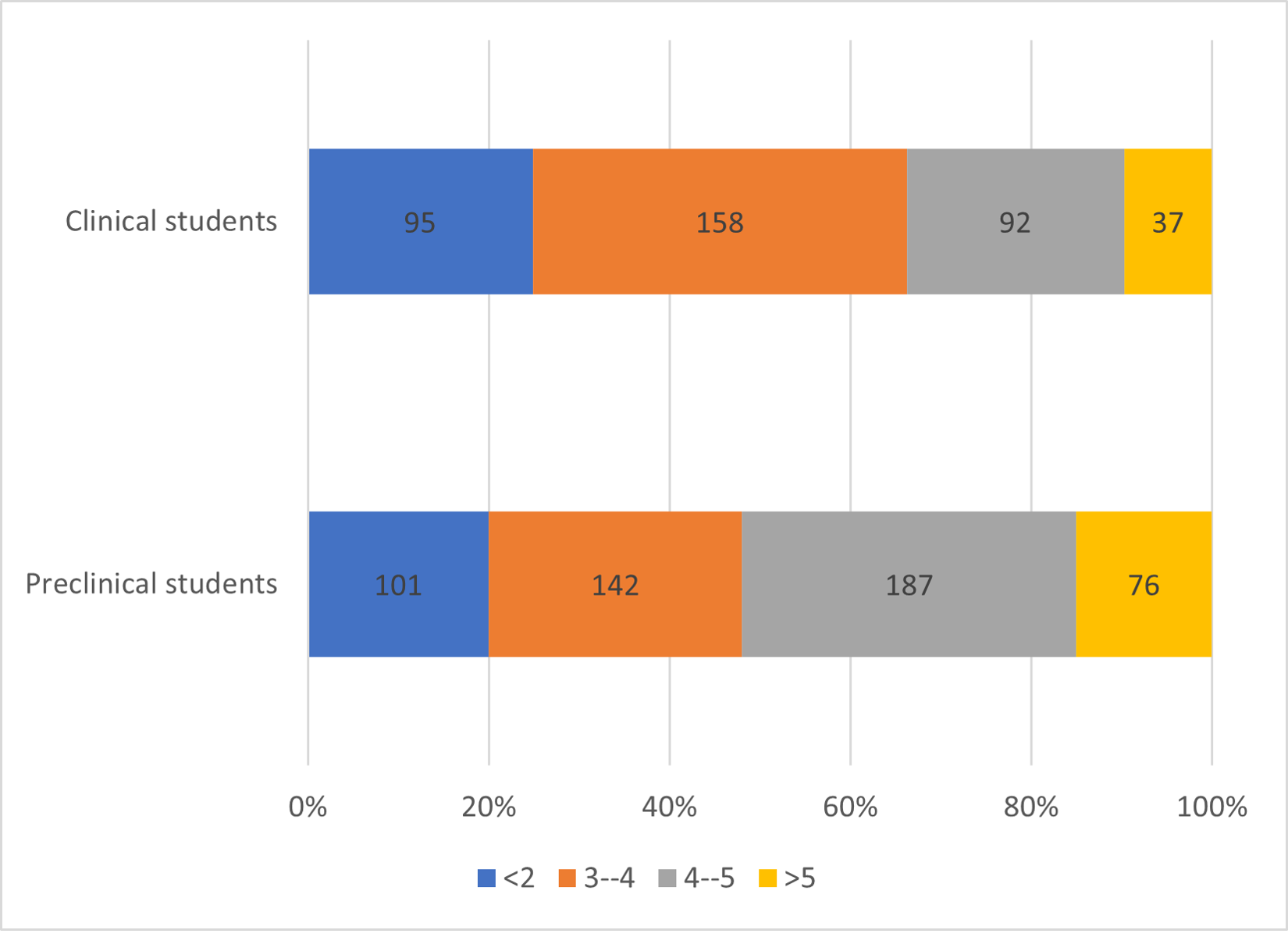

Supplement: S3 Fig — (TIFF) [file pone.0335664.s003.tiff]

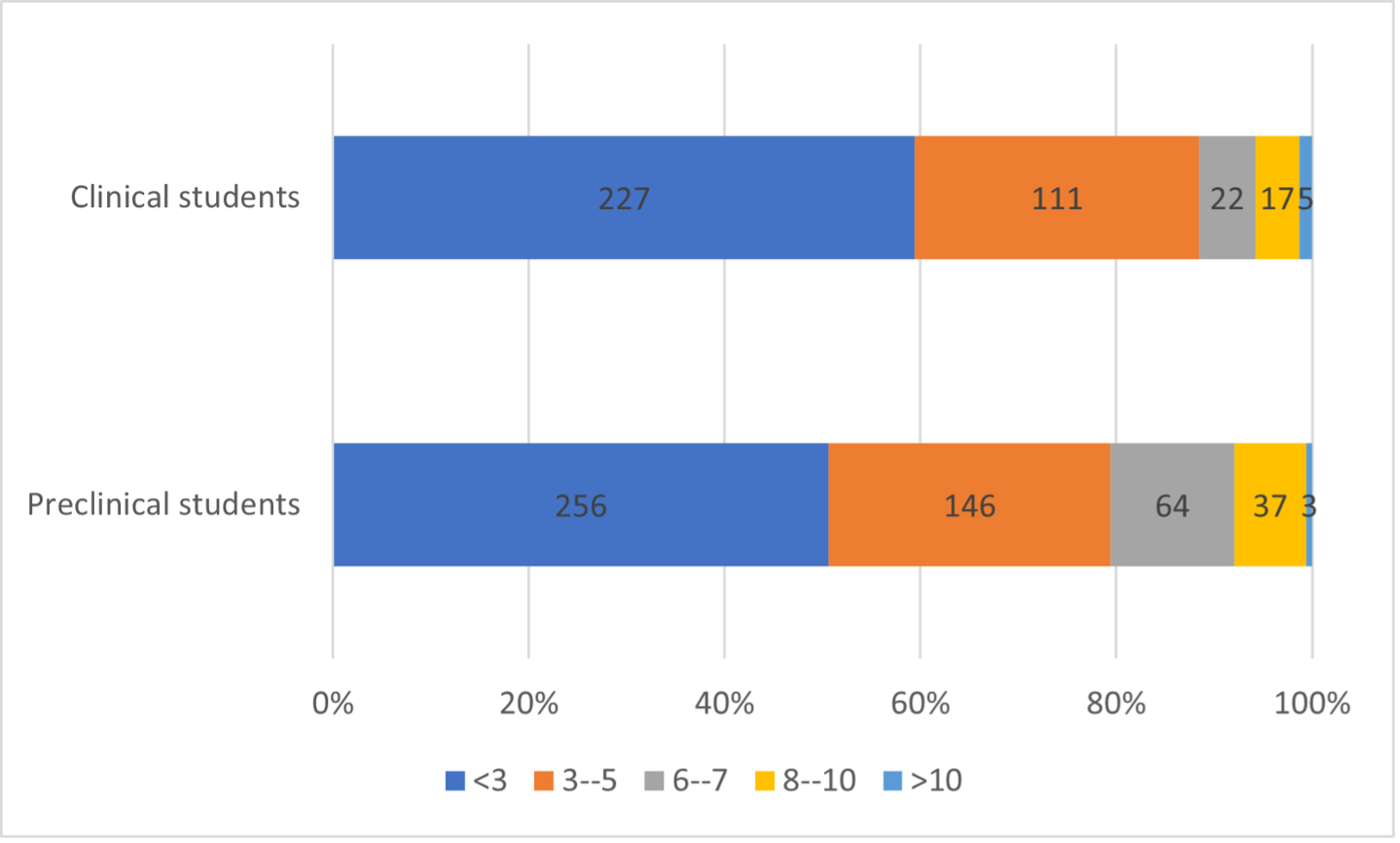

Supplement: S4 Fig — (TIFF) [file pone.0335664.s004.tiff]
